# Exploratory health economic analyses to support decisions within the innovation process in radiotherapy: Magnetic Resonance Linear Accelerator as a case study

**DOI:** 10.1016/j.tipsro.2025.100314

**Published:** 2025-05-14

**Authors:** Marike J. Ulehake, Ellen J.L. Brunenberg, Marcel Verheij, Janneke P.C. Grutters

**Affiliations:** a Science Department IQ Health, Radboud University Medical Center, Nijmegen, Netherlands (the); b Department of Radiation Oncology, Radboud University Medical Center, Nijmegen, Netherlands (the)

**Keywords:** Technology assessment, biomedical, Radiotherapy, image-guided, Quality-adjusted life years

## Abstract

**Background and purpose:**

There is an increasing need for understanding the added value of radiotherapeutic innovations before their widespread adoption. For multi-purpose innovations such as the Magnetic Resonance Linear Accelerator (MR-Linac), determining their value within a specific clinical context does not conclusively answer the broader question whether the innovation justifies the required investment. Exploratory analyses can be informative during early development and use of the innovation. We aim to develop and demonstrate an online, flexible tool to facilitate early economic evaluation of the MR-Linac.

**Materials and methods:**

We developed a tool that enables users to compare the total costs and incremental effects needed of MRI-guided radiotherapy (MRIgRT) with conventional treatment by inputting data specific to their context. Costs included in the tool are both medical technology and personnel-related expenses. The effects, required to justify potential additional costs, are expressed in Quality Adjusted Life Years (QALYs). By default, input values are used tailored to the Dutch clinical context.

**Results:**

When users input data specific to their context or situation, the tool generates figures that display the (additional) costs and effects of MRIgRT compared to conventional treatment. These figures enable users to explore the impact of key variables on the (additional) costs and QALYs required to justify any potential extra costs associated with MRIgRT.

**Conclusion:**

The tool and accompanying example allow for exploratory early HTA analyses, which can offer insights into the cost-effectiveness of MRIgRT. These insights can be valuable for guiding decisions regarding the purchase and utilization of innovative radiotherapeutic technologies.

## Highlights

• The tool allows stakeholders to explore MRIgRT’s costs and potential QALY gains.
• Examples demonstrate how the tool supports early economic evaluations of innovations.
• The tool provides insights into MRIgRT’s value for money and financial implications.

## Introduction

Radiotherapy is a rapidly evolving, technology-driven discipline [1]. Currently, the Magnetic Resonance Linear Accelerator (MR-Linac) is a promising innovation within the radiotherapy treatment repertoire. It integrates a magnetic resonance imaging (MRI) scanner and a linear accelerator. This technology, referred to as MRI-guided radiotherapy (MRIgRT), allows for plan adaptation using daily, soft-tissue visualization improving treatment accuracy and sparing surrounding healthy tissues [2].

Prostate cancer is a frequently chosen indication for MR-Linac treatment [3]. The use of MR-Linac is gradually expanding to other indications, such as lymph nodes, rectal tumors, and liver tumors, while evidence on the added benefit is still being collected [[3], [4], [5]]. This phenomenon of diffusion of the use of an innovation into patient populations, often without sufficient evidence of its effectiveness in these groups, is known as indication creep [6].

Furthermore, the MR-Linac requires significant investments for procurement, construction, staff training, and maintenance. Given the increasing pressure on sustainable healthcare systems, this raises important questions for hospitals: is the technology worth the investment, and, if a hospital has already adopted an MR-Linac, which patient groups should be prioritized for treatment?

Early Health Technology Assessment (HTA) offers methods, such as health economic modeling, to evaluate the (potential) value for money of new medical products throughout the innovation process, including research, investment, and development [7]. However, these models typically assess added value within a specific context and indication. For example, Schumacher et al. and Hehakaya et al., used health economic modeling to assess the cost-effectiveness of MRIgRT for prostate cancer [8,9]. While insightful for this clinical context, these studies offer limited guidance on optimal use of the MR-Linac across multiple indications (such as lymph nodes, rectal tumors, and liver tumors).

As the MR-Linac can be implemented in different healthcare settings and can be used for multiple indications, we propose a general technology-focused approach using exploratory health economic analyses to provide early insights in the (health) effects needed to compensate for the additional costs of using an MR-Linac, in order to be cost effective. We aim to demonstrate such analyses that support innovation and implementation decisions and we introduce an online, flexible tool to facilitate such analyses for stakeholders.

## Materials and methods

### Development of the tool

We designed a flexible tool for exploratory health economic analyses using the Shiny package 1.8.0 for R [10]. The web-based tool allows users to flexibly explore the (extra) costs and incremental effects needed for MRIgRT compared to conventional treatment. In the remainder of this article, we refer to conventional treatment as image-guided radiation therapy (without plan adaptation) on a linear accelerator with cone-beam CT. The tool intentionally does not target a specific indication or patient population upfront; instead, it is designed to be multi-purpose, allowing users to enter data for their specific situation of interest.

### Cost and effect calculations

The total treatment costs per patient include costs associated with the medical technology, personnel, and MR simulation costs (see Appendix A for formulas).

The costs for the medical technology include acquisition costs for the system and related equipment such as MR-compatible equipment for maintenance and QA, bunker construction, and yearly maintenance costs.

Annual costs were calculated by dividing the acquisition and construction costs by an annuity factor, accounting for the equipment's useful life years, along with the prevailing interest rate [11]. The annuity factor evenly distributes costs over the depreciation period. Separate factors were applied for medical equipment and the bunker, reflecting their specific schedules. Yearly maintenance costs were added to the annual costs for acquisition and construction. Subsequently, we calculated the price per minute of the medical device, based on the total annual costs and the hours per year the device is in use. The actual usage time is determined by multiplying the total of available minutes by the occupancy factor. The total costs for medical devices per treatment are calculated by multiplying the cost per minute by the number of fractions and fraction duration.

Total personnel costs per treatment include the time and type of personnel needed for preparation, execution, and, for MRIgRT, replanning. The total time for personnel equals the time per fraction multiplied by the total number of fractions and multiplied by the number of professionals present during treatment. This total time is then multiplied by the hourly of wage for the specific types of personnel (see Attachment B) [11,12].

Eventually, the cost for an MR simulation is added to the cost for the medical device and the cost for personnel, but only in the case of MRIgRT, as this cost is specific to the MR-Linac and would not be incurred in conventional radiotherapy. Costs common to both modalities, such as standard CT simulation, are not included, as they cancel out in a comparative analysis.

The cost difference between MRIgRT and conventional treatment – based on the given input – is calculated. To provide insight into the impact of key variables (the number of fractions, time per fraction, acquisition costs, total available time, and occupancy rate on the cost difference), the difference in costs and incremental effects needed is presented for a range of input values.

The incremental effects are expressed in Quality Adjusted Life Years (QALYs), a standard metric that combines quality of life and survival to assess disease burden. [13] This metric is used to quantify the potential benefits needed for MRIgRT. One QALY represents one year of life in perfect health. Depending on the additional costs of MRIgRT, the number of QALYs needed for MRIgRT to become cost-effective is calculated by dividing the additional costs by the cost-effectiveness threshold. For the base-case analysis, the cost-effectiveness threshold is set at 80,000 euros per QALY, which is the reference value for indications with a high burden of disease in the Netherlands [14,15].

### Default settings

To illustrate the tool, Dutch costs are used (Table 1). The cost are, where possible, derived from the Dutch costing manual [11]. Other default inputs reflect realistic estimates from Radboudumc’s clinical experience. It is assumed that both the conventional linac and MR-Linac operate at full capacity. As a default for the total available minutes, a total of 50 working weeks of 5 working days was assumed. Each day was estimated to consist of 9.5 working hours minus 135 min for maintenance, breaks, and other tasks. The total available time of the devices refers to the total time that the device potentially can be used for clinical procedures. Costs are presented in euros and indexed to January 2024 [16].

**Table 1:** Default input used in the tool.

Variable	Value	Source
*Costs for medical devices*		
MR-Linac treatment		
MR-Linac system	€9,500,000	Assumption based on the average list price in 2018
Bunker construction	€2,500,000	Assumption
Quality assurance or other related equipment	€0	Assumption
Annual maintenance costs	€665,000	7 % of the average list price
Maximum active time MR-Linac	1,813h	Assumption: 50 weeks of 5 working days of 9.5h. Minus 135 min per day for other tasks.
Occupancy rate	100%	Assumption
Time per fraction (minutes)	60	Assumption
Number of fractions per patient	5	Assumption
*Conventional treatment*		
Conventional system	€2,000,000	Assumption
Bunker construction	€500,000	Assumption
Quality assurance or other related equipment	€0	Assumption
Annual maintenance costs	€140,000	7 % of the average list price
Maximum time Linac	1,813h	Assumption: 50 weeks of 5 working days of 9.5h. Minus 135 min per day for other tasks.
Occupancy rate	100%	Assumption
Time per fraction (minutes)	15	Assumption
Number of fractions per patient	20	Assumption
*General*		
Annual interest rate	2.5%	Costing manual
Useful life years medical device	12	Assumption
Useful life years bunker	20	Assumption
*Costs for personnel*		
Hourly rate radiation oncologist	€105	Costing manual and collective agreement hospitals (2022/2023)
Hourly rate clinical physicist	€93	Costing manual and collective agreement hospitals (2022/2023)
Hourly rate radiotherapy technologist	€49	Costing manual and collective agreement hospitals (2022/2023)
*MR-Linac treatment*		
*Pre-treatment*		
MR simulation	€358	Costing manual
Total time radiation oncologist(s) (minutes)	135	Assumption
Total time medical physicist(s) (minutes)	15	Assumption
Total time radiotherapy technologist(s) (minutes)	210	Assumption
*Personnel present during treatment (number, and % of time available)*		
Radiation oncologist(s)	1 (20%)	Assumption
Clinical physicist(s)	1 (20%)	Assumption
Radiotherapy technologist(s)	3 (100%)	Assumption
*Conventional treatment*		
*Pre-treatment*		
MR simulation	€358	Costing manual
Time per radiation oncologist (minutes)	135	Assumption
Time per clinical physicist (minutes)	15	Assumption
Time per radiotherapy technologist(minutes)	210	Assumption
*Personnel present during treatment (number, and % of time available)*		
Radiation oncologist(s)	1 (20%)	Assumption
Clinical physicists(s)	1 (20%)	Assumption
Radiotherapy technologist(s)	3 (100%)	Assumption
*Costs for potential health effects*		
QALY	€80,000	Zwaap et al. (2015)

We emphasize that the Dutch costs and related assumptions used are for illustrative purposes only. The tool has been designed to be adaptable to different contexts, and users can modify the input values to reflect cost estimates and assumptions relevant to their own healthcare system (e.g., the U.S., other European countries, or any other context). This flexibility allows for context-independent use of the tool while ensuring its applicability to a wide range of settings.

## Results

### Description of the tool

Users can input their specific data to generate figures showing the costs and incremental effects needed of MRIgRT versus conventional treatment. The tool illustrates how variables like number of fractions, time per fraction, acquisition costs, available time, and occupancy rate affect cost differences and the additional QALYs needed to compensate MRIgRT’s additional costs, if any.

The tool is open-access and accessible via https://marikeulehake.shinyapps.io/Ulehake_MRL/.

### Examples of possible use

Below, we describe how the tool can be used by providing five illustrative examples, which are intended to show interested users the types of questions they can address with the tool.

#### Number of fractions

**Fig. 1:**
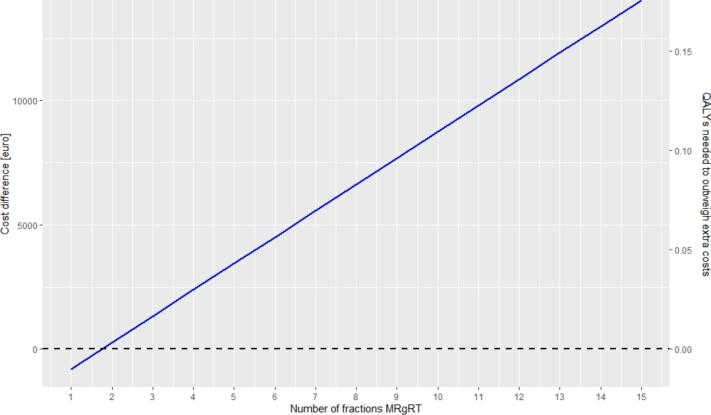
The impact of the number of fractions on the additional costs and the incremental effect needed for MRIgRT compared to conventional treatment. Default values as presented in Table 1 (including the cost-effectiveness threshold of €80,000 per QALY) are used for this figure, except for the number of MRIgRT fractions, which is varied between 1 and 15.

If users are interested in using MRIgRT for hypofractionation (providing the total dose in fewer treatment sessions), it is worth examining how the used treatment scheme affects the additional cost per patient. For example, with default inputs, the cost of MRIgRT is €1,942 per patient for a single fraction and rises to €18,079 for fifteen fractions. The cost for conventional treatment of 20 fractions of 15 min are €2,734 per patient. Resulting in a cost difference with MRIgRT ranging from €-793 for a single fraction (i.e., MRIgRT is less expensive) to €15,345 for fifteen fractions (i.e., MRIgRT is more expensive), with MRIgRT becoming more expensive with more than two fractions. If stakeholders accept additional costs, up to 0.19 additional QALYs per patient would be needed to justify the extra expenses, as shown in Fig. 1.

#### Time per fraction

**Fig. 2:**
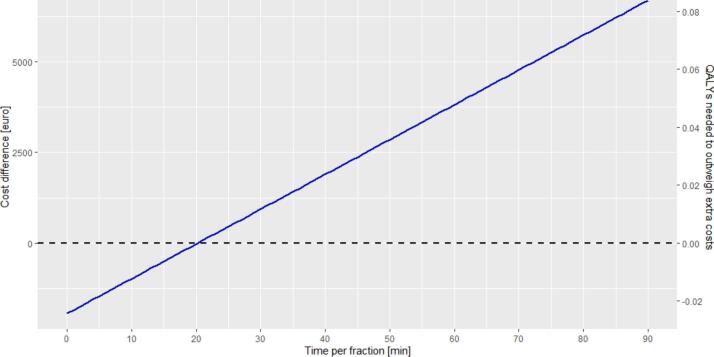
The impact of time per fraction on the additional costs and the incremental effect needed for MRIgRT compared to conventional treatment. Default values as presented in Table 1 (including the cost-effectiveness threshold of €80,000 per QALY) are used for this figure, except for the time per fraction of MRIgRT fractions, which is varied between 0 and 90 min.

The duration per fraction varies between patient groups. Users can explore how this impacts overall costs and the QALYs needed to justify them using the tool. With the default settings, treating a patient with five 30-minute MRIgRT fractions costs €3,671. Extending the fraction duration to 40 min increases the cost to €4,631. In comparison, using default values for conventional treatment with 20 fractions of 15 min costs €2,734 per patient (this is the difference between the total costs MR-Linac, and the difference compared to conventional treatment in the tool if default settings are used). Therefore, MRIgRT treatment of 5 fractions of 40 min is €1,897 more expensive, requiring a gain of 0.02 QALYs per patient to compensate the additional costs (Fig. 2).

#### Investment costs for the purchase of an MR-Linac

**Fig. 3:**
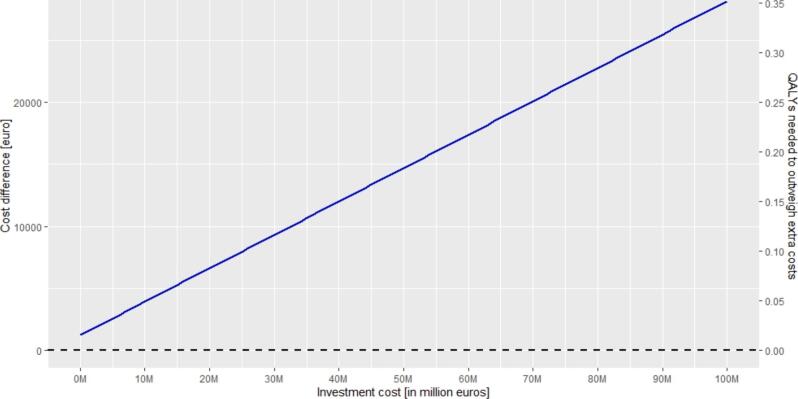
The impact of the investment costs for the purchase of an MR-Linac on the additional costs and the incremental effect needed for MRIgRT compared to conventional treatment. Default values as presented in Table 1 (including the cost-effectiveness threshold of €80,000 per QALY) are used for this figure, except for the investment cost, which is varied between 0 and 100 million euros.

For those considering the purchase of an MR-Linac, the tool helps explore how initial costs impact the additional expenses per patient and the additional QALYs required to justify these costs. If the MR-Linac and related equipment cost in total €10,000,000, and other default values apply, the tool indicates that approximately 0.05 additional QALYs per patient would be required to justify this investment (Fig. 3). Bunker construction costs are considered a separate item. Using the default values, even if the MR-Linac investment and related equipment were hypothetically provided at no cost, the cost per patient for MRIgRT treatment would still be cost more per patient due to other expenses, such as annual maintenance and bunker-related costs.

#### Utilization

**Fig. 4:**
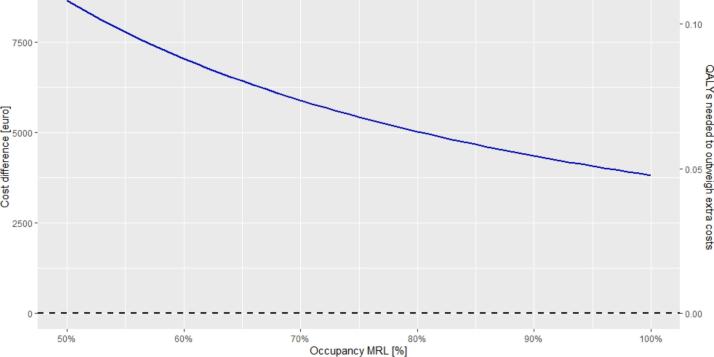
The impact of the occupancy rate on the additional costs and the incremental effect needed for MRIgRT compared to conventional treatment. Default values as presented in Table 1 (including the cost-effectiveness threshold of €80,000 per QALY) are used for this figure, except for occupancy of the MR-Linac, which is varied between 50% and 100%.

Users can explore the impact of MR-Linacs usages. If a hospital uses the MR-Linac for 70% instead of the full capacity due to time needed to be fully integrate the MR-Linac into the workflow, the additional cost per patient for MRIgRT compared to conventional treatment increases from €3,818 to €5,888 (Fig. 4). Consequently, the number of additional QALYs needed to justify these additional costs is 0.07 instead of 0.05 QALYs per patient.

**Fig. 5:**
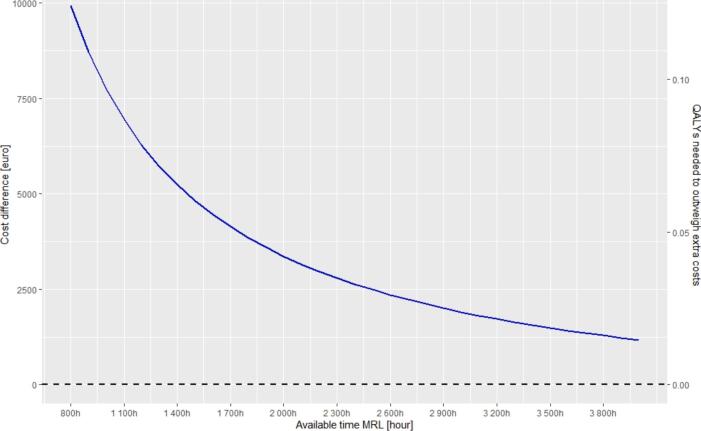
The impact of the total available time of the MR-Linac on additional costs and the incremental effect needed for MRgRT compared to conventional treatment. Default values as presented in Table 1 (including the cost-effectiveness threshold of €80,000 per QALY) are used for this figure, except for the available time of the MR-Linac for treatment, which is varied between 800 and 3800h a year.

Alternatively, extending MR-Linacs available yearly operating hours is an approach to increase patient capacity. For instance, with 2,000 annual hours, and the default treatment scheme of 5 fractions, each lasting 60 min, 400 patients can be treated per year per MR-Linac. In this scenario, MRIgRT would be €3,366 more expensive per patient compared to conventional treatment. To justify these additional costs, the tool indicates that 0.04 additional QALYs per patient would be required to offset the additional expenses. If the MR-Linac is available for 2,500 h, allowing 500 patients to be treated annually, MRIgRT would be €2,491 more expensive per patient requiring 0.03 additional QALYs per patient (Fig. 5).

#### Combining multiple variables

In the examples described above, one variable at a time was varied. In reality, a user may be interested in a combination of these parameters. For example, when comparing conventional treatment (10 fractions of 25 min) to MR-Linac treatment (40 min per session), the additional costs depend on the number of fractions given. With an MR-Linac costing 7.5 million, and both conventional linacs and MR-Linacs utilizing 100% of their 1,600 available hours annually and further using the default values, MRIgRT delivered in approximately 2 fractions is comparable in cost to conventional treatment. However, if delivered in 4 fractions, an additional 0.02 QALYs per patient would be needed to justify the extra cost of €1,358.

#### Clinical implications of required QALY gain

The previous analyses illustrate the number of QALYs needed in certain scenarios to offset the additional treatment costs. Such a QALY gain could for example be realized by increasing overall survival or improving quality of life by reducing toxicity. The feasibility of achieving such a reduction should be considered by expert opinion and, if available, clinical evidence. An example illustrating how to calculate the needed reduction in calculations based on the additional number of QALYs needed is provided in Box 1. Note, for simplicity, we did not account for the potential cost savings associated with preventing the complication. Therefore, the calculated reduction in complications may represent a slight overestimation.

Box 1 **Illustration of calculation the reductions in complications needed.**

For example, a urologist is interested to explore whether the additional costs of MRIgRT can be offset by the prevention of urinary incontinence. She finds in published literature that the disutility associated with urinary incontinence is 0.12 [17,18]. This means that over a 20-year period, a patient with urinary incontinence experiences a loss of 2.4 QALYs. The following calculation can be made: a prostate cancer patient who develops urinary incontinence after MRIgRT treatment—associated with a disutility score of 0.12 [17,18] over a 20-year period—experiences a loss of 2.4 QALYs. Therefore, preventing urinary incontinence in one patient would result in a gain of 2.4 QALYs. In one of the illustrated analyses (related to number of fractions), the QALY gain required to compensate for the costs is up to 0.19 for 15 fractions. To determine the necessary reduction in complications compared to conventional treatment, she calculates the complication risk needed to prevent by dividing the QALY gain per complication by the QALY gain per patient. In this example, this results in a reduction in complication of 7.9 % (0.19/2.4*100), depending on the number of fractions per treatment.

## Discussion

### Main findings

We constructed an online, flexible tool to demonstrate how exploratory early HTA analyses can offer insights into the cost-effectiveness of MRIgRT compared to conventional radiation treatment (using image-guided radiation therapy without plan adaptation on a linear accelerator with cone-beam CT). This tool is designed to explore the cost-effectiveness of the MR-Linac across multiple indications, making it particularly valuable for technologies that can be applied to a variety of clinical conditions. Its flexibility allows stakeholders such as purchasers and clinicians to enter data relevant to their specific situation and indication of interest. By helping stakeholders understand MRIgRT’s additional costs and the required effects to justify them, the tool provides support in procurement decisions and in determining appropriate indications for MR-Linac use.

### Interpretation of the results in relation to existing literature

The use of a flexible tool to provide insight in the cost-effectiveness of a (new) medical technology beyond a specific indication has been described before in the field of robot-assisted surgery. Patel et al. developed a tool aiming to estimate the potential benefits which are needed for a surgical robot to be cost-effective [17]. We applied similar methods within the field of radiotherapy specified for MRIgRT. As far as we are aware this is the first flexible tool to provide insight in the cost-effectiveness beyond a specific indication for MRIgRT.

Studies on the cost-effectiveness of the MR-Linac for prostate cancer treatment demonstrate its potential to be cost-effective compared to conventional treatment strategies [6,7]. Both Schumacher et al. and Hehakaya et al. used state transition modeling, wherein costs and effects are related to health states (e.g., no complications, complications, death). Direct comparisons with our results are not useful, as our research serves a different purpose and uses illustrative data. However, our analyses can help prioritize which indications warrant further investigations using advanced methodologies like transition modeling, which explores the potential cost and (long-term) effects more in-depth. The transition modeling approaches and the exploratory analyses we present can be seen as complementary approaches.

### Strengths and limitations

The R-IDEAL framework, designed to systematically evaluate innovations in radiation oncology, highlights the need to consider cost-effectiveness alongside implementation [18]. However, it lacks detailed guidance on the execution of (early) health economic studies. One of the strengths of our study is that it addresses this gap by providing a tool including illustrative examples of such analyses. Additionally, a key advantage of this tool is its adaptability; it can be used repeatedly in different healthcare contexts, over time and allows to be updated with new insights, making it valuable for ongoing decision-making.

A potential limitation of our study is the risk of users perceiving the tool as a black box, which may hinder their understanding and utilization. To address this, we provide a detailed description and examples of the analyses in this paper. In addition, the code of the tool is available via OSF.

Furthermore, users should note some limitations of the analyses itself. First, to enhance usability, we included only key cost drivers that are easily quantifiable and broadly applicable. As such, the tool does not include certain contextual and indirect factors, such as travel expenses, the total number of patients and their distribution across Dutch hospitals. Also, costs related to implementation, like additional training, required software or licenses and the learning curve, which may initially lead to longer treatment times, are not directly included. While these elements can influence cost-effectiveness outcomes, we aimed to balance completeness with usability. Therefore, the tool is designed to be adaptable, allowing users to adjust inputs such as treatment duration, occupancy rate and investment costs to indirectly explore the impact of these additional factors. Also, users can indirectly explore patient volume effects by adjusting the occupancy rate in the tool. Second, our focus was primarily on incorporating costs from a healthcare perspective, without assessing the reimbursement hospitals may receive from health insurers. As a result, the tool does not allow for drawing conclusion on a societal or hospital level. Future research could incorporate these broader perspectives. Third, the tool simplifies real-world complexities in clinical decision-making. While we evaluate value for money in terms of costs per QALY, purchasing decisions of costly medical technology should also consider strategic advantages of early adoption. For example, the model does not account for long-term cost savings resulting from reduced side effects or improvements in quality of life over time. Additionally, factors such as patient demand, patient availability, and ongoing research require careful consideration. To address these complexities, context-specific methods, such as transition modeling, may be better suited, highlighting the complementarity of our generic approach to inform context-driven methodologies.

### The role of early exploratory analyses for costly medical innovations

Our exploratory analyses are intended to complement more advanced HTA methods. These analyses should be conducted prior to using such methods or designing clinical studies, as they help prioritize which indications warrant further disease-specific investigation. Furthermore, the cost inputs collected for these early explorations can serve as a foundation for developing disease-specific health economic models.

With this article and the accompanying tool, we aim to stimulate early, informed dialogue among stakeholders to ensure that decisions regarding the adoption and implementation of MRIgRT are based on both clinical and economic considerations. Since clinicians and radiotherapy professionals play a critical role in the purchasing decisions for high-cost equipment [19], it is essential to inform them about the financial consequences associated with adopting MRIgRT. Our tool is designed to support this process by providing clear insights into the (potential) financial and clinical implications of MRIgRT adoption. It also helps stakeholders evaluate which patient populations or indications to treat, supporting discussions with healthcare insurers on the feasibility of integrating MRIgRT into existing treatment pathways. These early discussions are crucial, particularly in an era of rapid development of new, expensive medical technologies and growing healthcare expenditures.

While HTA may be perceived as judgmental due to its focus on evaluating healthcare interventions, the exploratory analyses presented here show that HTA can also provide valuable insights in initial discussions about the financial implications of MRIgRT. Although the tool was originally developed for MRIgRT, its underlying principles are also useful for informing discussions about the financial implications of other medical technologies within radiotherapy. The tool can be adapted to different radiation technologies by adjusting or adding relevant cost categories, such as device or personnel costs. If applied to another innovation, the core principles stay consistent: total costs are calculated from device and personnel costs, followed by cost per treatment, and the impact of key variable changes can be analyzed. However, significant adjustments are needed to tailor the tool to a new innovation, requiring proficiency in R Shiny.

## Conclusion

In conclusion, the tool and accompanying examples demonstrate exploratory early HTA analyses which can offer useful insights into the cost-effectiveness of MRIgRT. The analyses provide insight in the impact on costs and effects and can be valuable for guiding decisions, not only regarding the purchase of the MR-Linac but also regarding its exploitation.

## Declaration of Generative AI and AI-assisted technologies in the writing process

During the preparation of this work the author(s) used DeepL Write and ChatGPT to check for spelling and grammar and to improve readability. After using this tools/services, the author(s) reviewed and edited the content as needed and take(s) full responsibility for the content of the publication.

## Funding

This project is part of the PhD project of Marike J Ulehake and is funded by the Netherlands Organization for Health Research and Development (ZonMw) project number (10580012210015). The funding body has had no part in the study design, data collection, analysis, and writing the manuscript.

## Declaration of competing interest

The authors declare that they have no known competing financial interests or personal relationships that could have appeared to influence the work reported in this paper.

## Figures and Tables

**Table:** 

Costs of treatment per patient	Variable	Formula
*Total costs medical device*	Annuity factor medical devices =	(1/interest rate) * (1 − 1/((1 + interest rate)^useful life years medical device))
	Annuity factor bunker =	(1/interest rate) * (1 − 1/((1 + interest rate)^useful life years bunker))
	Total annual cost =	(Cost medical device + cost equipment for maintenance and QA)/annuity factor medical devices + (cost bunker construction/ annuity factor bunker) + annual maintenance costs
	Total cost per minute =	Total annual costs/max time medical device in minutes * occupancy rate medical device
	Total cost per patient =	Total costs per minute * (number of fractions * time per fraction)

*Total costs personnel*	Time involved in treatment* =	Total time pre-treatment in minutes + (time per fraction in minutes * number of treatment fractions * number of professionals present during treatment * % of time available during treatment)
	Costs personnel* =	Time involved in treatment * hourly wage/60 (professional dependent)
	Total cost per patient =	Cost radiation oncologist in minutes + cost medical physicists in minutes + cost radiotherapy technologist in minutes

*Cost MR simulation*		Cost for MR simulation

Total cost per treatment		Total cost personnel + total cost medical device + cost MR simulation
**QALYs needed to compensate for additional costs**		**(Total cost of MRIgRT per treatment − total cost of conventional treatment per treatment)**/**cost-effectiveness threshold**

**Table A.1:** Hourly wage based on the Cao (collective agreement) University Medical Centers 2022–2023 (salary 1-11-2023) [13].

	Radiotherapists (Pay scale 97A)	Clinical physicists (Pay scale 14)	Technologists (Pay scale 8 M)
Salary per month*	€11,932	€7,672	€4,228
Irregularity bonus 10 %*	€1,193	€767	€423
Social security contributions per month 35 %	€4,176	€ 2,685	€1,480
Subtotal per year	€207,614	€ 133,490	€73,570
Surcharge for job-related expenses	€6,866	€ 6,866	€0
**Subtotal per year**	**€214,480**	**€140,356**	**€73,570**
Workable hours per year	2,087	1,543	1,543
Cost per hour worked	€105	€93	€49

## Appendix A

Eq. A.1. Overview of formulas used in the tool to calculate treatment costs and required additional QALYs for MRIgRT cost-effectiveness.*Calculated separately for radiation oncologist(s), medical physicist(s), and radiotherapy technologist(s).

## Appendix B

*Middle of the indicated pay scale plus one.
